# Prevalence and determinants of smoking behavior among physicians in emergency department: A national cross-sectional study in China

**DOI:** 10.3389/fpubh.2022.980208

**Published:** 2022-10-17

**Authors:** Qiao Zong, Hui Li, Nan Jiang, Yanhong Gong, Jianwei Zheng, Xiaoxv Yin

**Affiliations:** ^1^School of Medicine and Health Management, Tongji Medical College, Huazhong University of Science and Technology, Wuhan, China; ^2^School of Health Services Management, Anhui Medical University, Hefei, China; ^3^Department of Social Medicine and Health Management, School of Public Health, Tongji Medical College, Huazhong University of Science and Technology, Wuhan, China; ^4^Department of Biliary-Pancreatic Surgery, Tongji Hospital, Tongji Medical College, Huazhong University of Science and Technology, Wuhan, China

**Keywords:** emergency physicians, smoking prevalence, title, depression, rotation, exercise

## Abstract

**Objectives:**

To understand the current status of smoking behavior among emergency physicians in China and to explore its determinants.

**Background:**

The emergency department is considered a more appropriate setting for tobacco interventions. However, the smoking behavior of emergency physicians can reduce the effectiveness of interventions for patient smoking behavior.

**Methods:**

From July to August 2018, we conducted a structured online questionnaire among Chinese emergency medicine physicians. We used descriptive analysis with binary logistic regression to analyze the current smoking status of Chinese emergency physicians and its determinants.

**Results:**

A total of 10,457 emergency physicians were included in this study. The prevalence of smoking among physicians was 25.35% (with 34.15 and 1.59% among male and female physicians, respectively). Results of logistic regression showed that postgraduate education (OR = 0.52, 95% CI: 0.41–0.66), chief-level title (OR = 0.79, 95% CI: 0.65–0.97), and regular exercise habits (OR = 0.83, 95% CI: 0.76–0.92) were associated with a lower risk of smoking behavior. However, being over 50 years old (OR = 1.71, 95% CI: 1.29–2.27), being fixed-term (OR = 1.25, 95% CI: 1.10–1.42), and having depressive symptoms (OR = 1.43, 95% CI: 1.28–1.61) were associated with a higher risk of smoking.

**Conclusion:**

The prevalence of smoking behavior among emergency physicians in China is high. Hospital management could reduce the incidence of smoking behavior among emergency physicians by strengthening smoking cessation training, paying attention to physicians' psychological health, reducing pressure on physicians in fixed-term positions, and encouraging physicians to develop regular exercise habits.

## Introduction

Tobacco use is a major public health concern in countries around the world (1). Smoking is responsible for more than 6 million deaths worldwide each year (2). Because physicians are the primary sources of health knowledge and medical advice (3), and their medical advice has a large impact on the population's health perceptions and health behaviors. The World Health Organization encourages and mobilizes health professionals to become actively involved in tobacco harm prevention and control (4). Studies have shown that physician interventions are effective and cost-efficient in improving patients' cessation behaviors (5). However, the presence of smoking among physicians not only directly affects physicians' attitudes toward tobacco control among smokers but also creates a false perception among their clients that smoking is harmless (6). Physicians who smoke are more likely to negatively counsel their patients to quit than those who do not smoke, thereby compromising the effectiveness of their interventions (7). Therefore, there is a need to understand the current status of smoking among physicians and to analyze its determinants.

Many studies have been conducted worldwide on the current status of smoking among physicians. The results of a meta-analysis that included 246 papers showed that smoking rates varied widely among physicians practicing different specialties (8). The smoking rate was 9% for radiologists, 18% for surgeons, 22% for internists, and 24% for family physicians. The source of the variation in smoking prevalence is closely related to the work environment and work stress in different departments (9). For example, the high frequency of night shifts, heavy workload, research pressure, and lack of team cohesion were identified as important risk factors for smoking behavior among physicians (10–12). In addition, previous studies have found that smoking behavior among physicians is associated with factors such as being male, divorced or separated, lower levels of education, depressive symptoms, and low levels of physical activity (6, 13–15).

Among the doctors in different clinical departments, the smoking behavior of emergency doctors should be focused on. On the one hand, most of the patients in the emergency department are acute and critically ill, and the work of the emergency department is characterized by complexity, variability, and suddenness (16). This leads to the emergency doctors' being often under great work pressure. There is a strong association between work stress and smoking behavior. On the other hand, it has been noted that emergency departments see a large variety of diseases and a large number of patients, and they are considered more appropriate settings for tobacco interventions than specialties with a single patient population (17). However, the effectiveness of tobacco interventions will be greatly reduced if emergency physicians engage in smoking behavior. A national survey of medical staff smoking behavior in the United States showed that emergency physicians had significantly higher smoking rates than physicians overall (5.7 vs. 3%) (18). An Irish study found that emergency physicians had the highest smoking rate of any physician surveyed, at 18.8% (19). These data suggest that the current status of smoking behavior among emergency physicians is not encouraging.

Although many surveys have been conducted on the prevalence of smoking among physicians in China, there is a lack of large-scale studies on the epidemiology of smoking among emergency physicians. In view of this, this study was conducted to understand the prevalence of smoking among emergency physicians in China and to identify the determinants, in order to provide an empirical basis for the subsequent improvement of physician tobacco control policies.

## Methods

### Ethics statement

Ethical approval for this study was obtained from the Research Ethics Committee of Tongji Medical College, Huazhong University of Science and Technology, Wuhan, China. Each participant was voluntary, and all identity details of study participants were kept confidential.

### Study design and data collection

This study is a national cross-sectional study. From July to August 2018, with the assistance of the Medical Administration Bureau of the National Health Commission of the People's Republic of China, a questionnaire and a link to the survey were generated through the online survey platform (platform name: Questionnaire Stra, website: http://www.wjx.com). The link to the survey was posted on the emergency physicians' work platform of the pre-hospital emergency facilities monitoring department, and all emergency physicians were invited to participate anonymously. The questionnaire was pre-tested with 30 emergency physicians to ensure that the questions were clear and understood by all participants. The survey link was re-posted to the workbench every 7 days to remind emergency physicians to take the survey until it was completed. All participants were required to read and agree to an electronic informed consent statement before completing the survey. All the data from this survey will be stored and managed on the “Questionnaire Star” platform.

A total of 15,288 emergency physicians clicked on the questionnaire link during this survey, of which 10,457 physicians validly completed the questionnaire, for a completion rate of 68.4%. Questionnaire entries included socio-demographic characteristics, work-related factors, personal health status, and whether they were smokers. Sociodemographic characteristics included gender, age, and educational background. Work-related factors included professional title, post, personal monthly income, frequency of night shifts, average number of trips per week, incidents of violence or patient verbal abuse experienced in the workplace in the past year, and job satisfaction. In addition, we investigated physicians' depressive symptoms, chronic disease and exercise habits.

### Definition

A “smoker” was defined as a person who has smoked at least 100 cigarettes or the equivalent amount of tobacco in their lifetime.

We used the job satisfaction scale in the Leiden Quality of Work Questionnaire to assess participants' job satisfaction (20). The scale contains six items on a 4-point Likert scale ranging from 1 to 4, indicating strong disagreement to strong agreement, respectively. The total scale score ranged from 6 to 24, with higher scores indicating higher levels of job satisfaction. The Cronbach's α of the scale was 0.85, indicating that the scale has good reliability.

We used the PHQ-9 questionnaire to assess participants' depressive symptoms (21). Items were scored on a scale ranging from 0 (“not at all”) to 3 (“almost every day”), with a total scale score of 0–27 for each participant, and the scale score was used to evaluate the respondent's depression. A score ≥10 was considered to indicate depressive symptoms. The Cronbach's α for the scale was 0.92, indicating that the scale has strong reliability.

### Statistical analysis

Continuous variables in descriptive analysis are expressed as means and standard deviations (SD), and categorical variables are expressed as frequencies and percentages. A *t*-test or ANOVA is used to compare differences between groups for continuous variables. The Chi-square test or Cochran-Mantel-Haenszel statistic will be used to compare whether categorical variables differ between groups. Finally, multifactorial logistic regression was used to identify risk factors for smoking behavior among emergency physicians and to give an adjusted OR value and 95% confidence intervals (95% CI) for each variable. Because of the small number of female physicians in the sample who smoked (only 45), our multifactorial analysis was conducted only for male emergency physicians. All comparisons were two-tailed. The significance threshold was set at a *P*-value < 0.05. All data analyses were performed using the Statistical Analysis System (SAS) 9.4 For Windows (SAS Institute Inc., Cary, NC, USA).

## Results

### Basic information and single factor analysis

The characteristics of the participants are presented in Table 1. The mean age of the physicians in this study was 36.53 (SD = 7.55), and male physicians accounted for nearly three-quarters of the physicians (72.98%, 7,632/10,457). The educational background of emergency physicians was concentrated on undergraduate degrees (74.49%, 7,789/10,457), with only one in ten physicians (9.41%, 984/10,457) having earned a master's degree or higher. In terms of mental health, 35.71% (3,734/10,457) of physicians suffered from depressive symptoms. And, almost half of the physicians did not exercise (46.83%, 4,897/10,457).

**Table 1:** Basic information of the sample and prevalence of tobacco smoking among Chinese emergency physicians.

	**Emergency physicians**		**Smoker**				***P*-value**
			**Yes**		**No**		
	***n***	**%**	***n***	**%**	***n***	**%**	
**Total (*****n*** **=** **10,457)**	**10,457**	**100**	**2,651**	**25.35**	**7,806**	**74.65**	
**Age (mean** **±SD)**	36.53 ± 7.55		37.42 ± 7.59		36.21 ± 7.51		<0.0001^*^
18–30	2,600	24.86	541	20.81	2,059	79.19	
31–40	4,977	47.59	1,308	26.28	3,669	73.72	
41–50	2,388	22.84	639	26.76	1,749	73.24	
>51	492	4.70	163	33.13	329	66.87	
**Gender**							<0.0001
Male	7,632	72.98	2,606	34.15	5,026	65.85	
Female	2,825	27.02	45	1.59	2,780	96.41	
**Education**							<0.0001
Junior college degree	1,684	16.10	541	32.13	1,143	67.87	
Undergraduate degree	7,789	74.49	1,940	24.91	5,849	75.09	
Master and Doctor degree	984	9.41	170	17.28	814	82.72	
**Level of hospital**							0.0393
Independent emergency center	1,681	16.08	463	15.60	1,218	17.47	
Grade 1 hospital	748	7.15	154	7.61	594	5.81	
Grade 2 hospital	4,442	42.48	1,181	41.78	3,261	44.55	
Grade 3 hospital	3,586	34.29	853	35.01	2,733	32.18	
**Job title**							0.6876
Resident	4,972	47.55	1,245	25.04	3,727	74.96	
Attending	4,112	39.32	1,068	25.97	3,044	74.03	
Chief	1,373	13.13	338	24.62	1,035	75.38	
**Job position**							<0.0001
Rotating shift	2,511	24.01	482	19.20	2,029	80.80	
Fixed shift	7,946	75.99	2,169	27.30	5,777	72.70	
**Monthly salary**							0.0400
<2,500 yuan	705	6.74	167	23.69	538	76.31	
2,500–6,000yuan	6,719	64.25	1,666	24.80	5,053	75.20	
6,001–10,000 yuan	2,693	25.75	735	27.29	1,958	72.71	
>10,000 yuan	340	3.25	83	24.41	257	75.59	
**Monthly night shift frequency**							<0.0001
0 times	494	4.72	95	19.23	399	80.77	
1–5 times	1,539	14.72	330	21.44	1,209	78.56	
6–10 times	5,633	53.87	1,400	24.85	4,233	75.15	
11–15 times	2,252	21.54	658	29.22	1,594	70.78	
>15 times	539	5.15	168	31.77	371	68.83	
**Times of following ambulance per week (mean** **±SD)**	14.75 ± 23.58		16.58 ± 26.11		14.13 ± 22.62		<0.0001
**Violence against doctors**							<0.0001
Yes	2,889	27.63	908	31.43	1,981	68.57	
No	7,568	72.37	1,743	23.03	5,825	76.97	
**Abused by patients**							<0.0001
Yes	8,555	81.81	2,271	26.55	6,284	73.45	
No	1,902	18.19	380	19.98	1,522	80.02	
**Job satisfaction (mean** **±SD)**	12.17 ± 3.58		11.73 ± 3.63		12.32 ± 3.55		<0.0001^*^
**Depress symptom**							<0.0001
Yes	3,734	35.71	1,170	31.33	2,564	66.67	
No	6,723	64.29	1,481	22.03	5,242	77.97	
**Chronic disease**							<0.0001
Yes	4,873	46.60	1,479	55.79	3,394	43.48	
No	5,584	53.40	1,172	44.21	4,412	56.52	
**Regular exercise**							0.0553
Yes	5,560	53.17	1,367	24.59	4,193	75.41	
No	4,897	46.83	1,284	26.22	3,613	73.78	

* This *p*-value is associated with *t*-test, all other *p*-values are associated with chi-square test or Cochran-Mantel-Haenszel Statistics.

In this study, the prevalence of smoking among emergency physicians was 25.35% (2,651/10,457) (Table 1), with 34.15% (2,606/7,632) and 1.59% (45/2,825) among male and female physicians, respectively (*p* < 0.01). Smoking prevalence differed significantly among other factors except for two variables, exercise and job title. Regarding educational background, the smoking rate among physicians with postgraduate and higher education was much lower than that of physicians with bachelor's or specialist's degree or less [17.28% (170/984) vs. 24.91% (1,940/7,789), and 32.13% (541/1,684), *p* < 0.01]. The highest prevalence of smoking was found among those aged 50 years or older and with a frequency of 15 or more night shifts, at 33.13% (163/492), and 31.77% (168/539), respectively. In addition, the prevalence of smoking among physicians with depressive symptoms was 31.33% (1,170/3,734). 55.79% (1,479/2,651) of physicians who smoke reported a history of chronic disease. It is of interest that physicians with these experiences had a higher smoking rate compared to physicians who did not experience violent incidents or verbal abuse by patients.

### Multifactor analysis

Table 2 demonstrates the results of a multifactorial logistic regression among male physicians in the emergency department. The results indicate that the risk of smoking increases with the age of the physician. Emergency physicians with a undergraduate degree (OR = 0.68, 95% CI: 0.59–0.78) and master and doctor degrees (OR = 0.52, 95% CI: 0.41–0.66) had a significantly lower risk of smoking than physicians with specialist degrees. Regarding work factors, chief physicians and fixed-term physicians were less likely to smoke. Depressive symptoms were associated with a 1.43 (95% CI: 1.28–1.61) unit increase in the risk of smoking behavior. Physicians with chronic diseases are 1.17 times (95% CI: 1.06–1.30) more likely to smoke than those with no previous history. The risk of smoking was 17% (95% CI: 0.76–0.92) lower among this group of physicians who exercised regularly each week compared to those who did not exercise.

**Table 2:** Multivariate logistic regression for characteristics associated with smoking behavior of male physicians in emergency department.

	**Tobacco use of Emergency**	
	**physicians**	
	**OR (95% CI)**	***P*-value**
**Age (ref** **=** **18–30)**		
31–40	1.18 (1.03–1.36)	0.0201
41–50	1.25 (1.04–1.50)	0.0190
50~	1.71 (1.29–2.27)	0.0002
**Education (ref** **=** **Junior college degree)**		
Undergraduate degree	0.68 (0.59–0.78)	<0.0001
Master and Doctor degree	0.52 (0.41–0.66)	<0.0001
**Level of hospital (ref** **=** **Independent emergency center)**		
Grade 1 hospital	0.90 (0.71–1.13)	0.3582
Grade 2 hospital	0.92 (0.77–1.06)	0.2446
Grade 3 hospital	0.90 (0.77–1.05)	0.1751
**Job title (ref** **=** **resident)**		
Attending	0.91 (0.80–1.03)	0.1324
Chief	0.79 (0.65–0.97)	0.0240
**Job position (ref** **=** **rotating shift)**		
Fixed shift	1.25 (1.10–1.42)	0.0005
**Monthly salary (ref** **=** ** < 2,500 yuan)**		
2,500–6,000yuan	1.04 (0.85–1.28)	0.6870
6,001–10,000 yuan	1.23 (0.99–1.54)	0.0671
>10,000 yuan	1.06 (0.75–1.49)	0.7373
**Monthly night shift frequency (ref** **=** **0)**		
1–5 times	0.90 (0.67–1.21)	0.4869
6–10 times	0.88 (0.67–1.16)	0.3705
11–15 times	0.92 (0.69–1.23)	0.5566
>15 times	0.84 (0.60–1.18)	0.3251
**Violence against doctors (ref** **=** **no)**		
Yes	1.02 (0.91–1.13)	0.7958
**Abused by patients (ref** **=** **no)**		
Yes	0.99(0.85–1.14)	0.8437
Times of following ambulance per week	1.00 (1.00–1.00)	0.1878
Job satisfaction	0.99 (0.98–1.01)	0.2565
**Depress symptom (ref** **=** **no)**		
Yes	1.43 (1.28–1.61)	<0.0001
**Chronic disease (ref** **=** **No)**		
Yes	1.17 (1.06–1.30)	0.0026
**Regular exercise (ref** **=** **no)**		
Yes	0.83 (0.76–0.92)	0.0004

## Discussion

This study found that 25.35% of emergency physicians in China have smoking behavior, with 34.15% of male emergency physicians. This is much higher than the smoking prevalence of emergency physicians in developed countries such as the United States (5.7%) and Ireland (18.8%) (18, 19). This may be related to the social environment and culture in China (22). In China, cigarettes are the preferred gift from patients to physicians, and physicians are accustomed to sharing cigarettes as a way to maintain social interaction with colleagues (23–25). Also, compared to physicians in other departments in China, emergency physicians have a higher smoking prevalence than the physicians in internal medicine, gynecology, and general practice (17.9, 7.7, and 7.3%, respectively) (26, 27), and also higher than the overall Chinese physician smoking prevalence of 20.4% (28). It has been suggested that emergency physicians work more frequently and under more pressure than other physician groups (29), which may lead to a higher prevalence of smoking behavior among emergency physicians.

Our findings show that the relationship between physicians' sociodemographic characteristics and smoking prevalence is similar to previous studies (28). The prevalence of smoking among physicians and the risk of smoking among physicians increased with age. This may be closely related to tobacco dependence and addiction resulting from a long-established smoking habit (30). In addition, smoking behavior showed significant gender differences. Male physicians had a much higher smoking rate than female physicians (34.15 vs. 1.59%), which is consistent with the vast majority of studies (8). To improve this, we can start by changing the stereotype that smoking is an act of masculinity, thus reducing the prevalence of smoking in the male emergency physician population and even in the general male population.

This study found that physicians with higher education and titles had a lower risk of smoking. In China, physicians with higher titles tended to have higher educational attainment (28, 31). The association between educational background and smoking behavior has been repeatedly mentioned in previous studies. The reason for the lower risk of smoking among physicians with higher education and higher titles is that, after a good medical education, physicians are more health-conscious, more health-literate, and have a fuller knowledge of the risks associated with smoking (32). As a result, they pay more attention to their health and actively avoid having such bad habits as smoking. Hospital administrators can organize regular health education seminars on tobacco cessation for emergency physicians with low education and titles to strengthen their awareness of tobacco cessation and improve their tobacco control skills by establishing the correct knowledge of tobacco in this group of emergency physicians.

In the emergency department, different work styles were also associated with smoking behavior. Our results show that physicians working on a fixed shift in the emergency department are at a higher risk of smoking than those working on a rotating shift. Emergency medicine physicians work in a setting that involves prolonged and frequent exposure to the possibility of witnessing death and trauma, diagnostic uncertainty, crowded access environments, and chaotic circadian rhythms (33). All of these factors make emergency physicians more likely to be at high risk for occupational stress. However, physicians on rotation usually work in the emergency department for only a few months and are less stressed and exposed to stress for a shorter period of time than fixed-term physicians, who face long periods of intense work and irregular work schedules. Therefore, the risk of developing smoking behavior is lower. This suggests to managers that the occupational stress of fixed-duty physicians should be appropriately reduced by rationalizing night shifts, for example, to reduce smoking behavior among emergency physicians.

Our study reaffirms the significant association between depression symptoms and smoking behavior. The present study found that physicians with depression symptoms were at a higher risk of smoking, which is consistent with the results of the literature review (34). The “self-medication hypothesis” suggests that smoking is a treatment modality that patients with depression choose to use to alleviate their symptoms (35, 36). In addition, the “false attribution hypothesis” also explains the relationship between depressive symptoms and smoking behavior (37). Withdrawal symptoms are similar to some of the emotional symptoms of depression, such as depressed mood, anxiety, and irritability (38). Cigarette smoking can alleviate such emotional symptoms caused by withdrawal symptoms, but smokers with depression symptoms attribute the ability of cigarettes to alleviate withdrawal symptoms to the ability of cigarettes to alleviate symptoms of psychological distress (39). This can exacerbate smoking behavior in patients with depressive symptoms. Therefore, we suggest that administrators regularly screen emergency physicians for mental health status and initiate timely psychological interventions for physicians who present with depressive symptoms.

From the results, it is clear that exercise habits are associated with a lower risk of smoking. Physicians with exercise habits may be more conscious of their physical health and may consciously refuse to smoke to avoid the physical harm caused by tobacco. At the same time, it has been suggested that exercise can reduce smokers' cravings for tobacco while increasing positive emotions to promote cessation (40). In this survey, only half of the emergency physicians had an exercise habit. This suggests that managers should encourage more staff to engage in physical activity and help them develop healthier lifestyles.

## Contributions and limitations of this study

Our study was the first to investigate the smoking status of emergency physicians and its determinants in 31 provinces nationwide to make a report, and is a national, large sample cross-sectional study. At the same time, our study has some limitations. First, as with other cross-sectional studies, this study could only determine correlations rather than causality, so we were unable to determine the order of appearance of chronic disease and smoking behavior, and future cohort studies with large samples are still needed to further elucidate the effect of emergency physicians' health status on their smoking behavior. Second, the definition of smokers in this study was based on the number of cigarettes ED physicians had already smoked, and their current smoking status was not investigated, so this study was not able to explore factors associated with ED physicians' current smoking status. And we did not focus on the physician's smoking cessation status or attitude toward quitting. The survey can be further improved in subsequent related studies.

## Conclusion

In conclusion, the prevalence of smoking behavior among emergency physicians in China is high. In order to give full play to the influence of emergency physicians in tobacco control initiatives and provide proper health guidance to patients, we need to develop targeted measures to improve the current smoking situation among emergency physicians and motivate them to make proper health decisions as soon as possible. Based on the results of the study, hospital administrators should focus on the smoking behavior of middle-aged, male, specialist-educated, junior and mid-level, and fixed-term emergency physicians. Measures should be taken to reduce the smoking rate among emergency physicians by enhancing smoking cessation training, focusing on physicians' mental health, reducing work stress among scheduled physicians, and encouraging physicians to develop regular exercise habits.

## Data availability statement

The original contributions presented in the study are included in the article/supplementary material, further inquiries can be directed to the corresponding author/s.

## Ethics statement

Ethical approval for this study was obtained from the Research Ethics Committee of Tongji Medical College, Huazhong University of Science and Technology, Wuhan, China. Each participant was voluntary, and all identity details of study participants were kept confidential. The patients/participants provided their written informed consent to participate in this study.

## Author contributions

YG, JZ, and XY contributed to the study design and data collection. QZ, HL, and NJ performed the data analysis. YG and XY supervised the data. QZ and HL contributed to the manuscript writing. All authors contributed to the article and approved the submitted version.

## Funding

This study was funded by the Department of Science and Technology of Hainan Province, Major Science and Technology Projects (ZDKJ202004) and Department of Science and Technology of Hainan Province, Key Research and Development Program (ZDYF2020112).

## Conflict of interest

The authors declare that the research was conducted in the absence of any commercial or financial relationships that could be construed as a potential conflict of interest.

## Publisher's note

All claims expressed in this article are solely those of the authors and do not necessarily represent those of their affiliated organizations, or those of the publisher, the editors and the reviewers. Any product that may be evaluated in this article, or claim that may be made by its manufacturer, is not guaranteed or endorsed by the publisher.
